# Distal Radial Access in STEMI: Where Feasibility Meets Clinical Reality

**DOI:** 10.1016/j.jacadv.2025.102474

**Published:** 2025-12-17

**Authors:** Ioannis Skalidis, Juan F. Iglesias, Grigorios Tsigkas, Mario Togni, Stephane Cook

**Affiliations:** a Department of Cardiology, HFR - Fribourg Kantonal Hospital and University, Fribourg, Switzerland; b Division of Cardiology, University Hospital Geneva, Geneva, Switzerland; c Department of Cardiology, University Hospital of Patras, Patras, Greece

Over the past decade, the transradial approach has supplanted transfemoral access as the default route for coronary intervention, offering superior safety and lower bleeding risk. More recently, distal radial access (DRA) has emerged as a promising evolution, potentially reducing radial artery occlusion and enhancing patient comfort. However, its role in acute coronary syndromes, where time and procedural efficiency are critical, remains under investigation.

In this context, Lee et al^1^ should be commended for conducting the DRAMI trial (Distal Radial Access vs Transradial Access in Patients With ST-Segment Elevation Myocardial Infarction), a multicenter randomized study comparing DRA and transradial access in patients with ST-segment elevation myocardial infarction undergoing primary percutaneous coronary intervention. Their work represents a timely and important contribution to the growing evidence base for DRA, offering one of the first randomized insights in this challenging clinical context.

Although noninferiority of DRA to transradial access for puncture success was not demonstrated in the intention-to-treat or per-protocol populations, the high success rate (94.3%) and comparable procedural outcomes across groups reaffirm the feasibility of DRA even in the emergency setting. The low rate of radial artery occlusion and similar bleeding complications further emphasize its potential safety advantages. These findings raise several questions that merit consideration in guiding future studies and clinical adoption.

First, could the learning curve and operator heterogeneity have influenced the apparent noninferiority gap? Despite inclusion of experienced operators, procedural nuances—such as puncture angle, ultrasound guidance, or side preference—might have affected early success rates. A standardized DRA training protocol or multicenter operator-proficiency analysis could clarify whether the residual difference reflects technique or anatomy.

Second, the smaller diameter and anatomical variability of the distal radial artery raise the question of optimal sheath size selection. Would the use of ultrasound-guided puncture, hydrophilic sheaths, or miniaturized equipment improve first-pass success and procedural comfort without compromising flow preservation?

Third, while the trial focused on puncture feasibility, the broader clinical question concerns long-term vascular preservation. Given the increasing emphasis on “access sustainability,” could future studies incorporate late vessel patency, functional flow assessment, and suitability for reaccess as endpoints? Understanding whether DRA translates to improved cumulative access-site health over time may redefine its clinical value beyond procedural success alone.

Finally, the slightly higher contrast use observed with DRA warrants exploration. Whether this reflects anatomic complexity, guide engagement challenges, or procedural ergonomics remains unclear.

The DRAMI trial sets an important benchmark for DRA feasibility in ST-segment elevation myocardial infarction and stimulates essential dialogue regarding training standardization, anatomical optimization, and longitudinal vascular outcomes. Addressing these questions may help establish DRA not merely as an alternative, but as a deliberate and sustainable strategy for coronary access in acute myocardial infarction.
